# Interpreting and validating complexity and causality in lesion-symptom prognoses

**DOI:** 10.1093/braincomms/fcad178

**Published:** 2023-06-05

**Authors:** Mohamed L Seghier, Cathy J Price

**Affiliations:** Department of Biomedical Engineering, Khalifa University of Science and Technology, Abu Dhabi, UAE; Healthcare Engineering Innovation Center (HEIC), Khalifa University of Science and Technology, Abu Dhabi, UAE; Wellcome Centre for Human Neuroimaging, UCL Queen Square Institute of Neurology, London, UK

**Keywords:** lesion-symptom mapping, prognoses, brain–behaviour relationships, probabilistic causality, logic

## Abstract

This paper considers the steps needed to generate pragmatic and interpretable lesion-symptom mappings that can be used for clinically reliable prognoses. The novel contributions are 3-fold. We first define and inter-relate five neurobiological and five methodological constraints that need to be accounted for when interpreting lesion-symptom associations and generating synthetic lesion data. The first implication is that, because of these constraints, lesion-symptom mapping needs to focus on probabilistic relationships between Lesion and Symptom, with Lesion as a multivariate spatial pattern, Symptom as a time-dependent behavioural profile and evidence that Lesion raises the probability of Symptom. The second implication is that in order to assess the strength of probabilistic causality, we need to distinguish between causal lesion sites, incidental lesion sites, spared but dysfunctional sites and intact sites, all of which might affect the accuracy of the predictions and prognoses generated. We then formulate lesion-symptom mappings in logical notations, including combinatorial rules, that are then used to evaluate and better understand complex brain–behaviour relationships. The logical and theoretical framework presented applies to any type of neurological disorder but is primarily discussed in relationship to stroke damage. Accommodating the identified constraints, we discuss how the 1965 Bradford Hill criteria for inferring probabilistic causality, *post hoc*, from observed correlations in epidemiology—can be applied to lesion-symptom mapping in stroke survivors. Finally, we propose that rather than rely on *post hoc* evaluation of how well the causality criteria have been met, the neurobiological and methodological constraints should be addressed, *a priori*, by changing the experimental design of lesion-symptom mappings and setting up an open platform to share and validate the discovery of reliable and accurate lesion rules that are clinically useful.

## Teaser

Seghier and Price framed causality probabilistically in lesion-symptom mapping, after defining 10 neurobiological and methodological constraints. Then, using the tools of logic and following the Bradford Hill criteria for inferring causality, they discussed the steps needed to generate pragmatic and interpretable brain–behaviour relationships in stroke survivors.

## Graphical Abstract

**Graphical Abstract:**
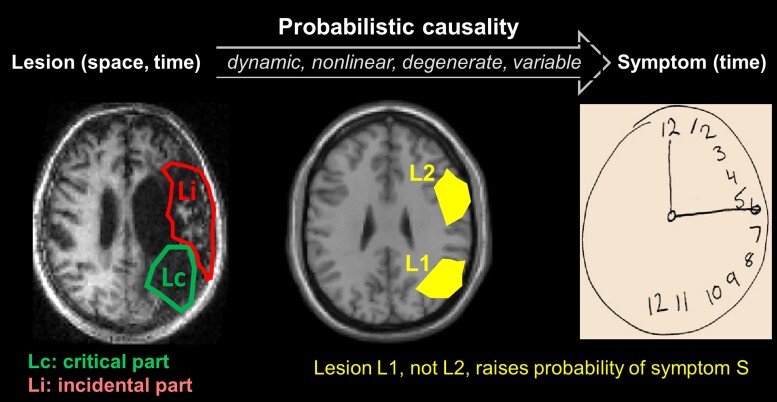

## Introduction

Here, we review a range of issues related to causality and validity in lesion-symptom mapping (LSM) and how such issues can be addressed when using neuroimaging data to generate valid and unbiased predictions of outcome and recovery following brain damage (i.e. lesion-symptom prognoses). Causation in LSM has been discussed and examined in previous reports (e.g. Godefroy *et al.*^1^, Toba *et al.*^2^, Siddiqi *et al.*^3^ and Sperber^4^) with an increasing appreciation of the importance of understanding the neurobiological mechanisms that influence how lesions produce symptoms. Validity, however, has been overlooked in this literature despite the fact that valid reasoning is critical for understanding the direction of causal effects and formulating predictions and prognoses.

Our paper builds on, and integrates, several lines of previous work. For example, Siddiqi *et al*.^3^ offered a multi-criteria continuum for assessing causality in findings from a range of brain imaging techniques and reported that LSM with naturally occurring lesions (e.g. stroke lesions) has moderate causality strength (a score of 4.5 out of 6.5 along a six-criterion scale) compared to other approaches, such as targeted brain stimulation. Other researchers have highlighted the graded nature of causation in complex biological systems where multiple factors contribute to the occurrence and severity of an effect.^5^ More generally, it has been proposed (e.g. Eells^6^, Mitroff and Silvers^7^, Sobel^8^ and Sprenger^9^) that it might be more intuitive to measure the strength of causal associations probabilistically; i.e. how likely it is that an effect will be associated with a cause.

Here, we go beyond prior reports by focusing on the logic and validity of causality in LSM. We consider the neurobiological and methodological factors that interact to produce a particular LSM outcome, unravel some inherent constraints that lessen the strength of causality in LSM findings, adapt prior criteria for assessing causality to make them relevant to the interpretation of lesion-symptom relationships and emphasize the need for probabilistic lesion-symptom prognoses. Although the theoretical framework discussed here applies to any type of neurological events that can cause a behavioural change, the majority of our examples concern the effect of stroke damage.

The organization of the paper is as follows: section ‘Causality and complexity in lesion-symptom mapping (LSM)’ describes how the deduction of causality in LSM is confounded by multiple levels of complexity that result in uncertainty and necessitate the formulation of probabilistic rather than binary associations. Section ‘The logic of combinatorial lesion-symptom mapping and its validity’ discusses how causal relations can be made stronger if supplemented by the application of logic and a deeper appreciation of the factors that determine validity. Section ‘Inferring causality, *post hoc*, along a multi-criteria continuum’ considers how causality can be deduced, *post hoc*, by applying a multi-criteria framework. Section ‘Implications for future LSM studies’ discusses potential implications for future LSM studies.

## Causality and complexity in lesion-symptom mapping (LSM)

LSM typically examines causal brain–behaviour relationships between a lesion L (focal damage or a multisite pattern) and one or more symptoms S from observational data. Lesion L is considered causal if, with all else held constant, its presence or absence affects the probability of observing a symptom S. Put another way, a direct causal relationship in LSM means that a change in L is sufficient to produce a change in S, without the operation of intermediate causes. A comprehensive framework is required to examine, interpret and validate the conditional statement L → S (where the arrow → indexes the direction of cause-and-effect). This section discusses how the application of L → S rules, and conclusions about causality, are confounded by multiple levels of complexity. Below, we identify five levels of complexity that arise from neurobiological constraints (NCs 1–5) and five levels of complexity that arise from methodological constraints (MCs 1–5) when making casual inferences at the group level.

**Figure 1:**
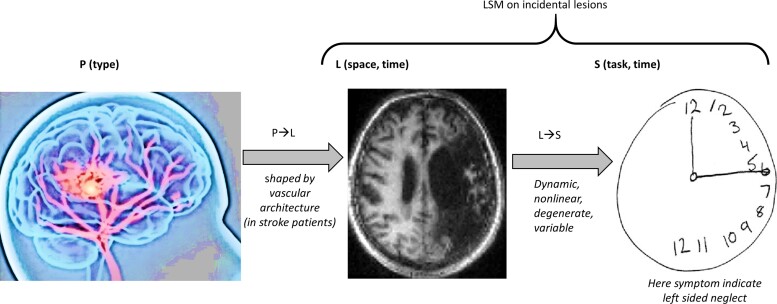
**Conditional relationships between pathology, lesion and symptoms.** An illustration of the causal relationships between pathology (P), lesion (L) and symptoms (S). The mapping from P to L is complex, shaped by the brain’s vascular architecture and dependent on space and time; type of pathology P can for instance be neurosurgery, ischaemic or haemorrhagic stroke, etc. The mapping from L to S is dynamic (varies with recovery/decline), nonlinear, governed by the rules of degeneracy and variable across patients and depends on many non-lesion confounders.

**Figure 2:**
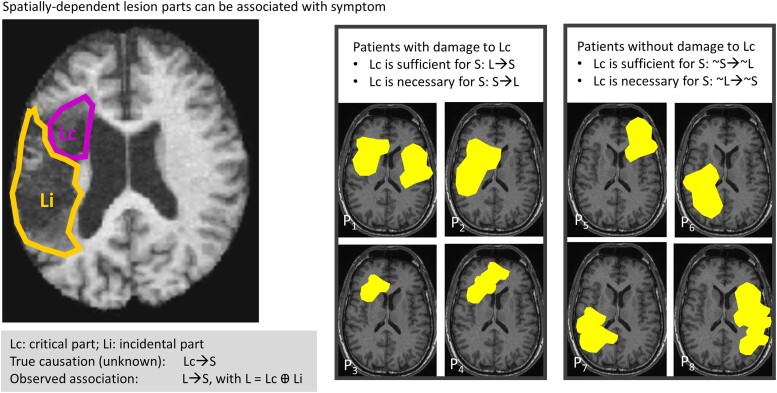
**Hypothetical spatially-dependent lesion-symptom relationships.** Left: each lesion is typically an aggregate of many small subregions or thousands of voxels, and it can be expressed at different spatial granularity. Here, the lesion site for a given patient is illustrated as a combination of (i) the critical part (Lc; a small polygone outlined in purple) that caused symptom S; and (ii) other incidental parts (Li; a large polygone outlined in orange) that did not cause the symptom but co-occurred with Lc (e.g. due to the vascular organisation of the brain). The full lesion is described as [Lc ⊕ Li] where [⊕] is the exclusive OR (xor) operator—meaning that Li is not part of Lc for symptom S. Li may, nevertheless, cause other types of symptoms and be further divided into Lc and Li for each of these other symptoms. The true causal relationship Lc → S is not known as the researcher can only assess the following relationship: [Lc ⊕ Li] → S. Right: critically, Lc cannot be distinguished from Li without establishing how different parts of the full lesion site [Lc ⊕ Li] are associated with symptoms in many other patients. To identify ‘sufficient’ brain structures, LSM (in large samples) is required to identify a candidate Lc region (set of voxels) that satisfies two criteria as follows: (i) ALL patients with damage to Lc have symptom S; i.e. [Lc_1_ → S] ᴧ [Lc_2_ → S] ᴧ [Lc_3_ → S], etc.; or (ii) ALL patients with no symptom S do not have lesion Lc; i.e. [(∼S → ∼Lc_5_)] ᴧ [(∼S → ∼Lc_6_)] ᴧ [(∼S → ∼Lc_7_)], etc. To identify ‘necessary’ brain structures, a candidate Lc region needs to satisfy two criteria as follows: (i) ALL patients with symptom S have damage to Lc; i.e. [S → Lc_1_] ᴧ [S → Lc_2_] ᴧ [S → Lc_3_], etc.; or (ii) ALL patients with no damage to Lc do not have symptom S; i.e. [∼Lc_5_ → ∼S] ᴧ [∼Lc_6_ → ∼S] ᴧ [∼Lc_7_ → ∼S], etc. It is expected that Lc is highly similar across patients with S (Lc_1_, Lc_2_, Lc_3_ … etc.), with differences within typical ranges of inter-individual variability in brain anatomy, whereas Li (Li_1_, Li_2_, Li_3_ … etc.) is expected to be highly variable across patients. Ultimately, the goal is to establish that: (i) Lc → S is true; and (ii) ∼S → ∼Lc is true, and Li → S is false. This is not possible in a single patient or a small sample of patients.

**Figure 3:**
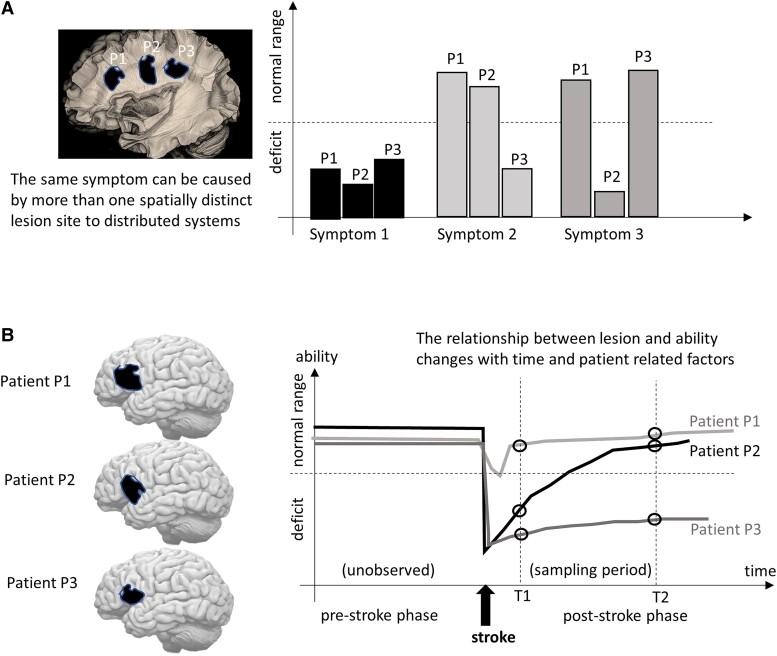
**Hypothetical patient-dependent and time-dependent lesion-symptom relationships.** (**A**) Three patients (P1, P2 and P3) with non-overlapping post-stroke lesions that severed different parts of the same white matter track. This results in the same deficit during one task (symptom 1) that relies on the damaged track, but different symptoms on other tasks that might reflect co-occurring damage to different regions. This example illustrates that lesions are associated with different behavioural profiles at a given time post-stroke. (**B**) Left: three patients P1, P2 and P3 with similar degrees of post-stroke damage to the left inferior frontal gyrus (damage indicated in black on the 3D brain images). Right: clinical scores (open circles) for a given ability (e.g. speech production) were collected at two different timepoints after stroke (T1 and T2). L → S is true for 66% of patients at T1 but only 33% at T2. This illustrates how inferences about causal relationships should ideally consider testing patients more frequently during the recovery period to track how brain–behaviour relationships change over time.

**NC-1:** spatial dependency—In humans, LSM deals with naturally occurring lesions that result from a particular pathology (Fig. 1) rather than an experimental intervention (as in animal models or with targeted stimulations). It is not possible to fully or tightly control for the location and extent of naturally occurring lesions because pathological lesions are constrained by other factors such as the vascular architecture of the brain,^10–12^ whether a stroke is ischaemic or haemorrhagic,^13,14^ and the type of neurodegeneration.^15^ When naturally occurring lesions are large or distributed, only a small part of the damaged region (lesion) may be causing the symptoms. We refer to this as the critical part of the lesion (Lc) and distinguish it from other parts of the lesion (Li) that are incidental to the symptoms of interest (Fig. 2). Separating Lc from Li is not a trivial problem because Li may be significantly correlated with symptoms when damage to Li is tightly correlated with damage to Lc.^11^ Spatial dependency refers to the correlation between the occurrence of Lc and the occurrence of Li.^12^ It results in the spurious correlation of Li with S, which contributes false positive effects to the results.
**NC-2:** distributed neural systems—It is well appreciated that motor and cognitive functions are supported by distributed sets of interacting brain regions that are referred to as neural systems, neural networks or neural circuits.^16,17^ If each function relies on multiple brain regions, then damage to any part of the neural system could theoretically disrupt function and cause the same symptoms (Fig. 3A). This can result in false negative results in group-level LSM experiments.^18^ For example, in a simple case where two different brain regions (X and Y) support the same function, patients identified by symptoms only may include a mix of those who have [X intact/Y damaged] and [Y intact/X damaged]—which results in high error variance, in both X and Y, when using univariate (e.g. voxel based) LSM.
**NC-3:** time-dependency—Even after a sudden neurological event, lesions continue to change over time in both the short-term^19^ and long-term.^20,21^ Symptoms also change with time in line with functional recovery (e.g. after stroke/neurosurgery) or functional decline in neurodegenerative disorders. The consequence of time-dependency in L and/or S is that significant L-S mappings may be observed at one timepoint but not another (Fig. 3B). In the case of stroke, for example, significant L-S mappings are more likely to be observed early post-stroke than after time has allowed for substantial plasticity, reorganization and recovery.^22–24^ Indeed, an increasing number of studies are showing that brain reorganization and recovery occur even years after stroke.^25–29^ Depending on the question and degree of recovery/decline, time-dependency will result in both false negative and false positive results in both univariate and multivariate LSMs.
**NC-4:** degeneracy in structure–function relationships—Lesion-symptom relationships may vary across patients when the same ability (function) can be sustained by different sets of brain regions (neural systems) in different individuals or at different times in the same individual. We refer to this type of pre-morbid inter-patient variability as ‘degeneracy’ and argue that it underpins one of the mechanisms that enable recovery of function after brain damage.^30,31^ The implication for group-level LSM (univariate or multivariate) is that degeneracy can result in false negative results because damage to the same region will be associated with symptoms in some patients but not others, depending on pre-morbid preferences for one neural system versus another.
**NC-5:** inter-individual variability in topography—Even after spatial normalization to a common template, there will be inter-individual variability in the topography of cortical regions and white matter tracts. For example, a particular brain region in one patient will not necessarily fall within a template parcellation for that region (see discussion in Bryce *et al*.^32^ and Moghimi *et al*.^33^). As with degeneracy, this variance could lead to false negative LSM results. This type of inter-individual variability is different from degeneracy (NC-4) that can arise not because of variance in the location of an anatomical structure, or its function, but in differences in how the functions are being recruited to perform a task either within patients in different contexts or between individuals.
**MC-1:** inaccurate lesion definition—In currently available brain scans, there is uncertainty in the definition of which parts of the brain are lesioned (noted L) or not lesioned (noted ∼L, where ∼ is the logical negation ‘not’). Consequently, some intact voxels might be misclassified as lesioned (∼L misclassified as L), and some abnormal voxels might be omitted from the lesion definition (L misclassified as ∼L). One way to minimize uncertainty in the definition of L and ∼L is to use continuous lesion definitions^18,34,35^ rather than the more commonly used binary lesion definitions (L or ∼L) adopted by most LSM studies. In addition to uncertainty in the definition of L, inaccuracies can also arise from suboptimal spatial warping of lesioned brains.^36,37^
**MC-2:** functional dysfunction—The absence of a visible/detectable lesion does not necessarily indicate healthy normally functioning brain tissue. Undamaged parts of the brain can have dysfunctional responses that contribute to symptoms and recovery because they are directly or indirectly connected to the lesioned area (cf. NC-2). In this context, symptoms can arise from remote dysfunction to different interconnected networks (e.g. ^38–40^). The possibility that parts of ∼L can also explain symptoms has ramifications for understanding validity and causality in LSM, as discussed in the next section.
**MC-3:** symptom complexity and severity—Symptoms are seldom a binary measure. They rely on a combination of observations from multidimensional data^41,42^ and can be expressed, across patients, with a wide range of severity. Consequently, a significant LSM might be observed at one level of symptom definition or severity but not another.
**MC-4:** context-dependency in LSM—Lesion-symptom relationships may vary within and/or across patients because the effect of brain damage on symptoms and recovery depends on multiple non-lesion variables such as age, co-morbid symptoms, self-motivation and type and frequency of therapy,^43–45^ which all need to be considered in LSM.
**MC-5:** functional specificity in LSM—The performance of any task rests on the combination of sensory, motor, attention and other functions. As many different tasks engage the same functions (e.g. domain-general systems^46,47^), damage to a shared function will result in multiple symptoms and affect multiple tasks. For example, damage to posterior inferior parietal regions can cause multiple symptoms in different domains.^48^ Interpreting lesion-symptom associations therefore requires consideration of how lesions affect performance across multiple tasks with shared and specific functions.

The awareness of the challenges associated with inferring causality is not new in the LSM literature. Seven decades ago, Lashley^49^ observed in animal models that behavioural deficits following brain lesions are frequently reversible, by alteration of input, context or additional brain lesions, implying a substantial degree of interaction between the distributed processing systems. The field at that time was converging to the following conclusion: the nervous system displays a level of interconnectedness and interdependence that makes it impossible to presume that a missing function is attributable to the removal of circuitry necessary for that function.

Grobstein^50^ appraised the work of Lashley and others and concluded that: ‘*it seems fairly clear that damage to one part of the nervous system is altering the characteristics of a distant part so that circuitry adequate to form a particular function is not expressed in behaviour*’ and therefore ‘*lesion analysis can be used to prove that a circuit is adequate to support a behavior but not that it is necessary*’. In terms of the neurobiological constraints discussed above, Grobstein’s conclusions are correct when there is time-dependency (NC-3), degeneracy (NC-4) or context-dependency (NC-5) in the LSM. However, there are also cases when it is possible to deduce the necessity of brain regions/circuits, for example, when the mapping between L and S is not dependent on time and does not vary across individuals. In line with Grobstein’s caution when inferring the ‘necessity’ of a brain region, we raise four additional points: first, if symptoms after a lesion only persist until surviving circuitry adapts to support recovery of intact behaviour, do we still refer to the lesioned brain region as ‘necessary’? Second, although a symptom/functional impairment may persist after small lesions to the brainstem, or corticospinal tract, naturally occurring lesions resulting in persistent impairments (i.e. are not subject to time or context dependency or degeneracy) are more likely to be those that are very large, making it difficult to determine which part of the lesion is necessary (NC-1 above). Third, concluding that a region is necessary for a ‘function’ does not imply that a lesion is necessary for a ‘symptom’ because when a neural circuit includes multiple regions, each of which is necessary (distributed neural systems), symptoms can be caused by multiple different lesion sites (NC-2 above). Finally, the term ‘necessary’ can be misleading when applying formal logic (as we do in the next sections) because identifying which regions are necessary does not imply that all other regions are not necessary.^51^ Given these challenges with the term ‘necessary’, we opt here to use the term ‘critical’ that simply implies that the lesion will have an effect on functional processing that might result in symptoms at some point after onset.

In summary, this section has defined multiple levels of neurobiological and methodological complexity in LSM that weaken the strength of the causal inferences that can be made from observational data. Causality can be defined as deterministic or probabilistic,^52,53^ but deterministic or true causation (L → S, L causes S) is extremely difficult to prove with LSM, if not impossible, due to the constraints outlined above. We conclude this section with the theoretical and practical implications for dealing with complexity in LSM and generating synthetic data.

Like others, we propose that LSM is best represented in terms of probabilistic causal relations^6–8^ that substitute the statement ‘L always causes S’ with ‘L’s occurrence increases the probability of S’, i.e. p(S|L) > p(S|∼L). Practically, this means that a lesion is considered more relevant to causality if it raises the probability of S.^9^ Given that naturally occurring lesions include multiple (critical and incidental) parts, the strength of causality needs to be considered separately for each part. This has been discussed in other domains under the framework of causal contribution^54^ that implies that a given site L_1_ is a stronger cause of S than site L_2_ if and only if L_1_ makes S more expected than L_2_. The implication for LSM is to consider L as a set of different parts with variable causality strength, varying from very strong in case of Lc to very low in case of Li of Fig. 2.

The probabilistic nature of brain–behaviour relationships, where the strength of causal associations varies with different parts of the lesion, also has implications for testing LSM with synthetic lesion data. Many studies^11,55,56^ have investigated the robustness of different LSM approaches using simulated data, considering the influence of spatial dependency (NC-1) and/or distributed neural systems (NC-2). The greater challenge will be to generate synthetic lesion data that simultaneously reflects all five neurobiological constraints and all five methodological constraints discussed in this section. By specifying these 10 criteria, we hope to motivate the generation of future synthetic lesion data that provides realistic explanations to black box findings.

Overall, by highlighting the constraints on LSM, our intention is to motivate users of LSM to (i) refrain from overstating ‘true’ causations; (ii) qualify the use of ‘necessity’; and (iii) adopt more pragmatic (and probabilistic) ways of understanding and inferring causality. We have also highlighted the need to distinguish between different types of L (Lc, Li, and ∼L) and how the relationship between L and S (L → S) should ideally be expressed as L(space,time) → S(time), see Fig. 1.

## The logic of combinatorial lesion-symptom mapping and its validity

Framing LSM in terms of logical symbols and notations is useful for clarifying, simplifying, validating and rigorously analysing the inferences that can be deduced from complex relationships. Logic can also help to identify and avoid fallacious reasoning, such as circular arguments. It can be used *post hoc* to evaluate existing observations and theoretical brain–behaviour relationships, and it is also expected to be useful for the *a priori* design of new experiments and for programming data-driven AI approaches to LSM. After reviewing the logical deductions that can be drawn from LSM, we highlight examples of when the application of logic is useful for clinical prognoses and formalizing theoretical frameworks.

Following the laws of conditional proof, LSM has typically been used to infer a ‘causal’ rule between lesion L and symptom S. The conditional statement, L → S, means that the presence of a lesion is sufficient to cause the presence of a symptom. It can be reframed in three ways: the logically equivalent contrapositive statement is expressed as ∼S → ∼L (if no symptom, then no lesion); the inverse is expressed as ∼L → ∼S (if no lesion then no symptom); and the converse is expressed as S → L (a symptom infers the presence of a lesion—which can only be the case when L is necessary to cause S).

In the context of distributed neural systems, when multiple distributed regions work together to support the same function (neurobiological constraint 2 in the previous section), the knowledge of all the possible lesion sites that have the potential to cause a symptom is not necessary to validate the conditional statement (L → S) or its contrapositive (∼S → ∼L). The statement [L → S] simply implies that L is one part of a distributed system associated with a symptom. Likewise, the statement [∼S → ∼L] simply implies that all critical lesion sites that have the potential to cause a symptom have been preserved. It is more difficult, however, to validate the converse [S → L] and inverse [∼L → ∼S] statements, because the presence of a symptom doesn’t imply the presence of a specific lesion site—it only implies damage somewhere in the distributed system. Likewise, the absence of a lesion site of interest (∼L) doesn’t imply the absence of a symptom (∼S) because the symptom could be caused by another lesion site. Therefore, in order to validate the statements S → L and ∼L → ∼S, we need to (i) identify all possible lesion sites associated with a symptom; and (ii) define ∼L as the preservation of all lesion sites associated with a symptom. This can be framed in Boolean terms using the operator conjunction (ᴧ) to imply that ‘all are present’; and the operator disjunction (v) to imply that either one or another is present. For example, if a neural system includes regions X and Y, the (disjunctive) expression L_x_ v L_y_ is used to indicate that damage to either X or Y (or both) can yield the symptom. In other words, the statement S → L can be re-expressed as [S → (L_1_ v L_2_ v L_3_ v … L_n_)]; and the statement ∼L → ∼S can be re-expressed as [(∼L_1_ ᴧ ∼L_2_ ᴧ ∼L_3_ ᴧ ∼L_n_) → ∼S].

Degeneracy (neurobiological constraint 4 in the previous section) can also be expressed in Boolean terms. For example, when a function of interest can be supported by either region X or region Y, the (conjunctive) expression [(L_x_ ᴧ L_y_) → S] can be used to indicate that the ‘lesion’ associated with a symptom covers two regions, X and Y, and lesions to both are required to cause the symptom. Hence, the absence of a symptom implies that at least one region is preserved and able to maintain function. In our example of a two-region system (X and Y), this contrapositive can be expressed as [∼S → (∼L_x_ v ∼L_y_)]. In other words, only X or only Y is damaged: [(L_x_ ᴧ ∼L_y_) → ∼S] or [(L_y_ ᴧ ∼L_x_) → ∼S]. It is also possible to combine different logical operators when describing degeneracy in distributed system, for example [(L_1_ v L_2_) ᴧ (L_3_ v L_4_) → S] indicates that symptoms will be observed if there is damage to L_1_ or L_2_ in combination with damage to L_3_ or L_4_. Another interesting example describes paradoxical lesions^57^ when, in the context of L_x_, a symptom is not observed if L_y_ is also present [(L_x_ ᴧ ∼L_y_) → S] ᴧ [(L_x_ ᴧ L_y_) → ∼S].

Spatial dependency (neurobiological constraint 1 in the previous section), when multiple functional regions are damaged by the same lesion, will result in combinations of different behavioural symptoms i.e. [L → S_1_ ᴧ S_2_ ᴧ S_3_ ᴧ … S_n_]. The precision of this statement can be improved when the lesion can be divided into multiple parts. Ideally, we need to partition the lesion into parts that are critical or incidental to specific symptoms of interest. For example, [Lc → (S_1_ ᴧ ∼S_2_)] and [Li → (S_2_ ᴧ ∼S_1_)]. When the critical and incidental parts are unknown, under investigation and or being refined, lesions can be divided into different parts using anatomical parcellations.^58,59^ However, as anatomical parcellations do not correspond to either functional regions or the spatial distribution of lesions goverened by pathology, it may be more informative to segregate lesions in a data-driven way.^18,41,60,61^

Time-dependency (neurobiological constraint 3 in the previous section) can also be factored into combinatorial rules. This is essential to the clinical application of [L → S] rules that are only relevant for predicting outcome after brain injury when they are specified in time. For example, the statement [L → (S_T1_ ᴧ ∼S_T2_)] indicates that a lesion site will result in temporary symptoms at time 1 but not time 2 (due to recovery at time 2); the statement [L → (S_T1_ ᴧ S_T2_)] indicates that the symptoms will persist at time 2 as well as time 1, and the statement [L → (S_T2_ ᴧ ∼S_T1_)] indicates that symptoms will emerge at T2 (e.g. decline in neurodegenerative disorders).

Our example of a clinical application concerns [∼L → ∼S] rules in pre-surgical mapping for the treatment of epilepsy and brain tumours. Here, the question is whether cortical stimulation (a temporary induced lesion) to site L causes symptom S. If so, the surgeon may avoid resection (∼L) to minimize post-surgical impairment (∼S). Critically, however, preserving L (∼L) cannot be used to imply the absence of symptoms when other areas are being resected. Inferring ∼S will only be possible once the full set of possible L sites associated with S is known, and the following statement is validated [∼L_1_ ᴧ ∼L_2_ ᴧ ∼L_3_ ᴧ ∼L_n_ → ∼S]. A second example of a clinical application for lesion-symptom rules is when the presence of a symptom is used to predict the presence of a lesion [S → L]. For example, after the discovery that damage to Broca’s area caused speech production difficulties (Broca’s aphasia), some researchers assumed that Broca’s aphasia was the consequence of damage to Broca’s area. However, all that can be inferred for symptoms associated with distributed systems is that some part of the system has been damaged. Neuroimaging data is required to indicate which part of the system is damaged (unless damage to different parts can be inferred from unique combinations of symptoms).

Finally, framing LSM in logical combinatorial rules provides a powerful and grounded way to test existing theoretical ‘modes’ describing different types of brain–behaviour relationships. Table 1 represents the brain modes previously considered.^1,2^ We have supplemented these modes and their definitions by adding: (i) the equivalent logical rules for each mode to allow researchers and clinicians to frame their hypotheses and test the validity of combinatorial lesion rules when assessing lesion-symptom relationships; and (ii) the implications for recovery. Overall, the tools of logic can be extremely helpful to ensure valid inferences from LSM by examining the soundness of lesion rules and the associated brain–behaviour modes.

**Table 1:** Logic rules and recovery predictions for each of the theoretical ‘modes’ for brain–behaviour relationships

Godefroy *et al*.^1^ and Toba *et al*.^2^		Logic rules, validity and recovery predictions (this study)	
Mode	Definition	Rule (logic)	Recovery prediction
The unicity mode	One single lesion (L) causes the symptom (S).	L → S	Recovery potential is very limited. This mode mainly applies to lesions along primary or sensory pathways.
The equivalence mode	Lesions to either structure x (L_x_) or y (L_y_) cause symptoms (S).	(L_x_ v L_y_) → S	Recovery speed depends on the learning capacity of the spared (undamaged) regions.
The association mode	Both L_x_ and L_y_ must occur to cause symptoms (S).	(L_x_ ᴧ L_y_) → S	Slow recovery when both lesions are present. No or mild deficit when only one lesion is present.
The summation mode	Symptoms (S) are observed after L_x_ or L_y_ (equivalence) but are worse when L_x_ and L_y_ co-occur (association).	[(L_x_ ᴧ L_y_) → S] ᴧ [(∼L_x_ v ∼L_y_) → ∼S]	Slow recovery when both lesions are present. Faster recovery when only one lesion is present.
The mutual inhibition/masking summation mode	L_y_ paradoxically reduces S caused by L_x_.	[(L_x_ ᴧ ∼L_y_) → S] ᴧ [(L_x_ ᴧ L_y_) → ∼S]	Recovery from L_x_ is faster when L_y_ is induced by brain stimulation.

The first two columns are definitions of ‘modes’ taken from Godefroy *et al.*^1^ and Toba *et al.*^2^

## Inferring causality, *post hoc*, along a multi-criteria continuum

A nuanced approach for considering causality is to assess it along a multi-criteria continuum. For example, the Bradford Hill framework includes nine criteria to consider when inferring causality from correlation as follows: (i) strength; (ii) consistency; (iii) specificity; (iv) temporality; (v) biological gradient; (vi) plausibility; (vii) coherence; (viii) experiment; and (ix) analogy; see below. This approach has been widely adopted in epidemiology to shift experimental inference from correlation to causation. Theoretical applications of this framework have been adapted for the assessment of causality in correlations between functional connectivity and post-stroke recovery^62^ and between repetitive head impacts and chronic traumatic encephalopathy.^63^ Traditional LSM does not meet all these criteria (see Siddiqi *et al*.^3^ for a full discussion). Here, we re-frame the same set of nine Bradford Hill criteria (referring to them as BH-1 to BH-9) for assessing causality in lesion-symptom relationships, inter-relating them with the five neurobiological constraints discussed in the first section above.

### BH-1: a high correlation between brain lesion and symptoms, across patients (strength)

An essential first step in the identification of a potentially causal relationship is to demonstrate a high correlation between brain lesion and symptoms, across large samples of patients. This helps to focus on areas with low inter-patient variability avoiding the challenge of high inter-patient variability (NC-4 and NC-5 in the first section above). Although the strength of a lesion-symptom relationship can be used as a proxy for the soundness of causal relationships, this is limited. High correlations may be spurious, particularly in small sample sizes that are vulnerable to outliers and not representative of the population^64^ or when there is spatial dependency of damage^12^ across functionally unrelated brain regions (NC-1 in Section I). Conversely, weak correlations may still reflect a causal relationship, for example, when patients are tested at different timepoints during their recovery or decline (NC-3 in Section I) and/or when the same symptom can be generated by different lesion sites, within the same neural circuit (NC-2 in Section I).

### BH-2: lesion load (biological gradient)

A causal relationship between lesion and symptoms is more likely when it can be demonstrated that the severity of symptoms increases with the amount of damage to a critical region—just as the degree of therapy-induced improvement is expected to be related to the amount (dose) of therapy. This has been shown in the many studies that identified: significant relationships between deficit severity and lesion load,^65,66^ the degree of damage to critical sites,^60^ the lesion impact score to major brain hubs^67^ or the degree of disconnection to key white matter tracts.^58,68^ The greater the lesion load, the more likely it is that there is damage to one or more critical parts of the lesion (Lc in Fig. 2). Notably, however, high lesion load does not necessarily indicate more severe symptoms, particularly when a region of interest includes parts that are differentially related to the function (NC-1 in Section I). For example, if only a small part of the lesion is critical to a function (Lc), the rest being incidental co-occurring damage (Li), then high lesion load could be observed without the symptom so long as the critical part (Lc) is spared.

### BH-3: lesion site dependent symptoms (specificity)

Causality is more probable when symptoms can be shown to relate to one lesion site more than another (or one part of a lesion site more than another). This can be expressed as p(S|L) > p(S|∼L), i.e. the conditional probability of observing S is higher when L is present than when L is absent. A second type of specificity occurs when a lesion-symptom relationship has time-dependency (NC-3 in Section I) because symptoms may be observed in the acute phase after a neurological event but not in the chronic phase when recovery is more likely to have occurred.

### BH-4: lesion before symptoms (temporality)

If the lesion caused the symptoms, then the symptoms will occur after the lesion. Put another way, damage always precedes the onset of a symptom. This is easy to deduce after neurological events like stroke but may be less easy to infer in neurodegenerative conditions. It also applies to inferences about recovery, with the expectation that recovery will follow neuroplasticity/brain reorganization irrespective of whether improvement was spontaneous or therapy-induced. Ideally, a demonstration of temporality requires longitudinal, prospective studies (within patients). Alternatively, the time course of recovery can be estimated from assessments staggered over time across patients,^69^ or self-reported descriptions of the time course of an individual’s recovery from patients, their family or friends.

### BH-5: reproducibility (consistency)

A causal relationship between lesion site and symptoms should be observed across patients, studies, pathologies (e.g. damage caused by stroke or neurosurgery) and investigators. Demonstrating this (i.e. cross-validation) can be across heterogeneous samples and methodologies (indicating a universal relationship) or in groups of patients with specific demographics or treatments when context dependency needs to be controlled (MC-4 in Section I).

### BH-6: indirect reproducibility/consistency (analogy)

This is the demonstration that an association mimics other causal relationships that have already been demonstrated. For example, in the context of distributed neural systems (NC-2 in Section I), the effect of damage to one part of a neural circuit is expected to have a similar effect as damage to a second part of the same neural circuit.^62^ Therefore, if a lesion to one part of a white matter tract has previously been proven to be causal, then the evidence required to infer a causal association is slightly lowered, by analogy, when symptoms are observed following damage to another part of the same white matter tract.

### BH-7: experimental manipulation

While it is possible to generate probabilistic lesion-symptom relationships from naturally occurring lesions, causal relationships can be made more compelling if supplemented by external interventions.^70^ This can be expressed as follows: p(S|ex(L)) > p(S|ex(∼L)), where the ex(L) means to experimentally intervene on L, (i.e. an active manipulation of L) and the expression overall means that L is a cause of S if and only if an intervention on L changes the probability that S has a particular deficit severity. For example, in the context of spatial dependency (NC-1 in Section I), experimental lesions (e.g. induced surgically in animals or virtually with neurostimulation or pharmacology in humans) might be able to distinguish which part of a lesion is causing the symptoms and which part of a lesion is spuriously associated with the symptoms.

### BH-8: biologically sensible lesion-symptom relationships (plausibility)

A causal relationship is more likely if it fits with prior observations, current scientific knowledge and established theory. For example, there is plausibility when observing visual perceptual impairments following damage to parts of the well-recognised visual processing pathways. This criterion fits well with a more Bayesian view of LSM in which prior knowledge is used to evaluate different models and explanations.

### BH-9: not biologically implausible (coherence)

A causal relationship doesn’t necessarily have to replicate established knowledge but it should not profoundly conflict with what is already known from other fields and approaches.^63,71^ An example of a lack of coherence would be if LSM associated damage to the visual cortex with auditory rather than visual perceptual impairments. This does not mean that findings that do not align with established knowledge should be ignored. It means that extra effort should be taken when checking the validity of surprising findings particularly when LSM is based on purely data-driven approaches. ‘Coherence’ has also been defined as ‘the convergence of different approaches that reach similar results’,^3^ which blends the ideas of coherence (BH-9) with consistency (BH-5).

## Implications for future LSM studies

Applying the nine Bradford Hill causality criteria to LSM results, *post hoc*, allows us to shift our inferences from correlation to causation.^62^ The number of criteria that are met, along with a priority system (e.g. temporality is mandatory, whereas experimental manipulation is rarely possible), allows different degrees of causal inference to be ranked.^3^ The goal of LSM is to maximize the likelihood that the causal parts of a lesion (Lc) are dominating the lesion-symptom relationship (Fig. 2).

For clinically reliable lesion-symptom relationships, validation needs to satisfy at least one of the following two criteria, in at least two independent datasets as follows: (i) a high positive predictive value [i.e. L → S, the probability that a patient with lesion L has a positive (abnormal) test result (symptom S)]; and/or (ii) a high negative predictive value (i.e. ∼L → ∼S, the probability that a patient is symptom free at a particular timepoint in recovery following a lesion that spares all the lesion sites that have previously been associated with that symptom). These requirements are not met by current LSM methods because of challenges related to (i) segregating Lc from Li (NC-1 in Section I); and (ii) defining ∼L that requires *a priori* knowledge of all possible lesion sites that have the potential to cause symptoms (see Sections II and III).

Given these constraints and limitations, it might make sense to take a more pragmatic approach by: (i) relaxing the assumption of a strong causal relationship at every identified region in LSM, and shifting interest towards the discovery of useful lesion rules; and (ii) replacing the emphasis on ‘ALL patients’ meeting a lesion-symptom rule with probabilistic estimates that aim to show ‘the expected proportion of patients’ that will meet a lesion-symptom rule. The higher the probabilistic relationship, across patients, the higher the likelihood of a strong causal lesion; and the more confident we can be in generating accurate prognoses for new patients. Furthermore, it is also essential that LSM findings are integrated with findings from other techniques to ensure convergence on common neuroanatomical circuits.^72–74^ Overall, by unravelling these constraints, we hope to help make sense of LSM findings that do not always converge with findings from other brain mapping techniques (cf. BH-5 and BH-9).

## Conclusion

When assessing causal relationships with LSM, observational data brings many challenges. Lesions (causes) are not manipulated by the experimenter, and it is not possible to undo the impact of the lesion on the vascular architecture or to control brain reorganization over time. In this paper, we have (i) defined five neurobiological and five methodological constraints on LSM; (ii) examined LSM through the lenses of logic; and (iii) considered how causality can be inferred from correlations through the nine Bradford Hill causality criteria. The Bradford Hill criteria are useful for assessing the strength of causality *post hoc*. We now propose that rather than rely on *post hoc* evaluation of how well the causality criteria have been met, it might be possible to address the neurobiological and methodological constraints, *a priori*, by making the LSM approach more pragmatic. This requires changing the experimental design of LSM, a possibility that will be addressed in future work. As LSM is increasingly used on diverse clinical groups, we call for an open platform to share the identified lesion rules (e.g. as 3D maps of critical lesions in a stereotaxic standard space) so that their explanatory and predictive power can be tested in new samples. The expectations here is that sharing LSM-based lesion factors will speed up the discovery of reliable and accurate lesion rules that are clinically useful.

## Data Availability

Data sharing is not applicable to this article as no new data were created or analysed in this study.

## Abbreviations

BH =: Bradford Hill
L =: lesion
Lc =: critical part of a lesion
Li =: incidental part of a lesion
LSM =: lesion-symptom mapping
MC =: methodological constraint
NC =: neurobiological constraint
S =: symptom
